# Mechanical Behavior of Honeybee Forewing with Flexible Resilin Joints and Stripes

**DOI:** 10.3390/biomimetics8060451

**Published:** 2023-09-24

**Authors:** Dan Hou, Zheng Zhong

**Affiliations:** 1 Department of Mechanical Engineering, Shanghai Maritime University, Shanghai 201306, China; dhou@shmtu.edu.cn; 2 School of Science, Harbin Institute of Technology, Shenzhen 518055, China

**Keywords:** resilin joints and stripes, honeybee forewing, flexible airfoil, modal dynamic analysis

## Abstract

The flexibility of insect wings should be considered in the design of bionic micro flapping-wing aircraft. The honeybee is an ideal biomimetic object because its wings are small and possess a concise vein pattern. In this paper, we focus on resilin, an important flexible factor in honeybees’ forewings. Both resilin joints and resilin stripes are considered in the finite element model, and their mechanical behaviors are studied comprehensively. Resilin was found to increase the static deflections in chordwise and spanwise directions by 1.4 times and 1.9 times, respectively. In modal analysis, natural frequencies of the first bending and first torsional modes were found to be decreased significantly—especially the latter, which was reduced from 500 Hz to 217 Hz—in terms of resilin joints and stripes, closely approaching flapping frequency. As a result, the rotational angle amplitude in dynamic responses is remarkable, with an amplification ratio of about six. It was also found that resilin joints and stripes together lead to well-cambered sections and improve the stress concentrations in dynamic deformation. As resilin is widespread in insect wings, the study could help our understanding of the flexible mechanism of wing structure and inspire the development of flexible airfoils.

## 1. Introduction

The flight of insects has attracted much attention in many fields, especially that of insect-mimicking flapping-wing micro air vehicles (FMAVs). FMAVs refers to aircraft that can fly at low speed and at various altitudes, like birds and insects, and achieve hovering, forward, and backward flight with high maneuverability and low power consumption [1]. The size of FMAVs is generally defined as being less than 15 cm, and their mass as less than 100 g. In terms of comprehensive performance, FMAVs are still far from being able to fly as freely as insects. One of the main reasons is likely because the mechanical structures are always designed rigidly, while the biological material and structures found in nature are soft and flexible. Therefore, it is necessary to explore the flexible mechanism as well as deformation behaviors of insect wings and consider them in the bionic design of FMAVs.

Experiments have shown that there are obvious deformations, including twisting and bending, in the wings of insects such as dragonflies, butterflies, and bees during flight [2,3]. Through wind tunnel testing and numerical calculation, the two forms of deformation have been proven to directly influence flight aerodynamics [4,5]. Firstly, a wing with its base constrained twists like a propeller during each stroke to meet a similar angle of attack along a spanwise direction, which is helpful in high lift production. Secondly, the wing is always cambered to reorient the aerodynamic force direction and enhance wake capture effects. In insects, during flight, deformations of the wing far from the base are considered to be induced passively. Both morphological structures and materials play significant roles in transmitting forces and movements in this process. This passive deformation mechanism needs to be clearly understood.

Previous researches found that resilin—generally, the resilin-bearing vein joints—was found to contribute a lot to the chordwise flexibility of the wings of insects such as dragonflies, beetles, and bumblebees [6,7,8,9,10]. Although some of the conclusions are based on speculation, the functions of resilin on insect wings have attracted much attention. Resilin is a polymeric rubber-like protein which can be stretched to over 300% of its original length before breaking and possesses a low elastic modulus, in the range of 0.1–3 MPa [11,12]. Mountcastle and Combes [10] demonstrated, by experiment, that the chordwise stiffness could be enhanced by 36% when a central resilin-bearing vein joint was fixed artificially. Later, they found that a resilin-bearing vein joint at the leading edge helped wasp wings to avoid damage in collisions [13]. In addition, resilin is considered to provide and store sufficient elastic strain energy to reduce the wing damage in Odonata [14,15] and periodically help wing-folding in beetles [16] and Asian ladybirds [17]. Inspired by the resilin-bearing joints in dragonfly wings, biomimetic timber joints were developed, and their high performance was analyzed [18]. As the morphology and distribution of resilin joints in insect wings are very complicated, most of the studies focus on a single joint without considering the effects on the overall wing structure.

In addition to the general resilin-bearing vein joints, there are resilin stripes in areas where the longitudinal vein and membrane are connected in some insect wings. Ma et al. [19] found comparable resilin joints and stripes in honeybee wings by experiment and suggested the existence of five flexion lines in one forewing–hindwing entity, which could probably increase chordwise flexibility. Compared with resilin joints, the functions of resilin stripes in insect wings have not received much attention. In this paper, the roles of both resilin joints and stripes are considered in the honeybee forewing. Due to the irregular shape of resilin patches and numerous vein joints, building a very precise finite element wing model with resilin is complicated. Thus, our finite element model was built in a relatively simplified way but fully considering the flexible effects of resilin. On one hand, the longitudinal and cross-veins were connected by connectors defined with hinge properties to simulate the function of resilin joints in transmitting torques. On the other hand, longitudinal veins and the membrane were connected with constraint, allowing relative rotation to simulate the function of resilin stripes. Deformation and dynamic responses were analyzed and compared through different models to determine the roles of resilin joints and stripes on the mechanical behaviors of honeybee forewings.

## 2. Materials and Method

### 2.1. Resilin in Honeybee Forewing

**Figure 1:**
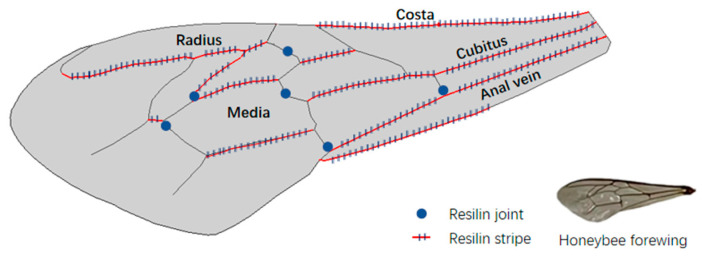
Distribution of resilin is sketched from a real honeybee forewing. The resilin joints distribute on the cross-veins and, therefore, these cross-veins are deemed to be cut off by them. The resilin stripes are distributed at the connecting region between the veins and membrane.

Honeybees are small and excellent at hovering. Their maximal wingspan is about 1 cm. The wings of a honeybee include forewings and hindwings, and the latter are much smaller. In this paper, the forewing is considered in analysis. The wing base is quite narrow and connected with the thorax muscle and it is supported mainly by the robust longitudinal veins in front, namely, the costa, cubitus, and anal veins. Compared with insects such as dragonflies, honeybees have more concise vein venation and much fewer vein joints. Veins in the central area are radius and media veins, which form several joints and cells with irregular shapes. The posterior edge is soft and not completely wrapped by veins. The wing surface is relatively flat, and the small vein corrugations were not considered in the model for lack of precise measurements. As shown in Figure 1, distributions of resilin joints and resilin stripes were precisely marked according to the experimental results [19]. There are six resilin-bearing vein joints, and three of them are located in the middle area. Furthermore, one joint is in the front, and two joints are close to the midpoint of the chord length near the wing tip. Resilin stripes are found along longitudinal veins except for some radiuses near the wing tip. The content of resilin at vein joints and stripes is quite small, according to observation of fluorescent microscopy images (Figure 5 in [19]). In addition to the two forms of resilin, there is resilin concentrated at the wing base, with higher content in insects such as honeybees. This makes sense, because the base is the pivot in a flapping motion with high frequency, and material of high elasticity and resistance to fatigue is needed.

### 2.2. Finite Element Models

#### 2.2.1. Materials and Geometry

**Figure 2:**
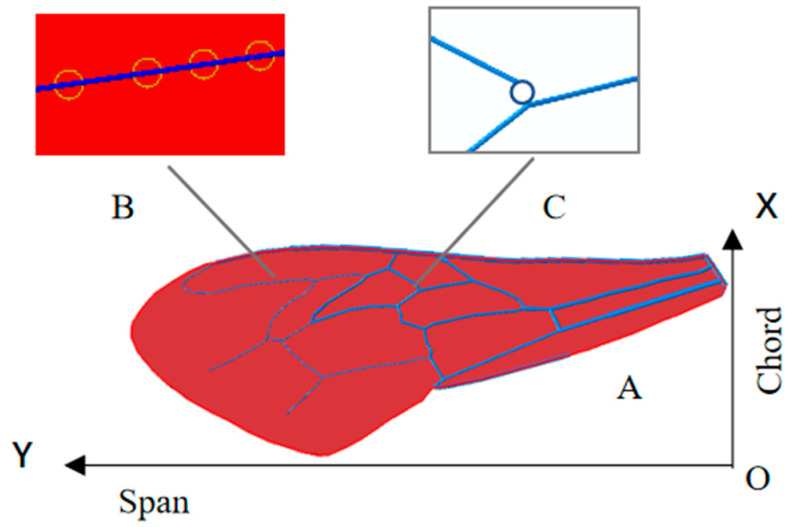
Finite element model of honeybee forewing (A) with vein-membrane connecting stripes (B) and a hinged vein joint (C). The XOY frame is fixed on the wing with its origin at the root. X is the chordwise direction, pointing from trailing edge to the leading edge; Y is the spanwise direction, pointing to the wing tip.

The finite element model was built from a photo of a honeybee forewing in the ABAQUS software, as shown in Figure 2. The wingspan was 9 mm and the maximum chordwise length was 3 mm. The wing structure is mainly composed of veins and membrane. The veins are of a three-dimensional circular tube structure, which was simulated by a two-node beam element, B31. According to the SEM measurements [20], four diameter sizes were set in the model to distinguish the veins’ distribution in different areas, as shown in Table 1. The membrane was extremely thin, with approximately uniform thickness. It was found that the in-plan tensile stress could stiffen the membrane in the transverse direction [21]. Therefore, the four-node thin shell element S4R was appropriate for simulating the membrane. Veins and membrane were tied together to obtain overall wing structure. This constraint ensures that the two parts are not separated during analysis. Parameters in geometry and material were determined and shown in Table 1, referring to some experimental measurements [20,22,23].

#### 2.2.2. Modeling of Resilin Joint and Stripe

The huge difference in material properties between resilin and chitin was brought into focus. Young’s modulus of vein and membrane constituted by chitin are 4.8 GPa and 2.8 GPa, which is thousands of times higher than that of resilin (0.1–3 MPa). Therefore, the area connected by resilin is considered to be very soft and flexible compared with other areas. This justifies the following simplifications in the finite element model. Firstly, the resilin joints were built by setting the vein junctions as hinged. Secondly, the resilin stripes were simulated by allowing the relative rotation of the nodes in vein-membrane adjacent elements. By using these approaches, the flexible effects of resilin are fully considered in the models. Details such as the unsymmetrical distribution of resilin in dorsal and ventral sides were not taken into account in the modeling.

#### 2.2.3. Loads and Boundary Conditions

The wings of honeybees always flap at high frequencies in flight, and the main loads are borne by inertia and aerodynamic force. The higher frequency the wing flaps, the more significant effects the inertia produces. Flapping motion is similar to fixed-axis rotation, and the constraints and motions are all applied on the wing base in the following analysis. Due to the structure flexibility and inertial force produced in dynamic flapping under the given motion, deformation of the wing can be observed. According to previous researches [24,25,26], the flapping motions of insect wing can be characterized by flapping angle φ(t) and torsional angle α(t). φ(t) is the angle of rotation around the *X*-axis and α(t) is the angle of rotation about *Y*-axis, as shown in Figure 2. They are defined as:

(1) $$\begin{array}{l} \varphi (t)=-\frac{\varphi_{\max}}{2}\cos2\pi ft+\frac{\varphi_{\max}}{2}\\ \alpha (t)=\frac{\pi }{2}-(\frac{\pi }{2}-\alpha_{\min})\sin2\pi ft \end{array}$$

where the maximal flapping angle $\varphi_{\max}$ and minimal torsional angle $\alpha_{\min}$ are $131^{\circ }$ and $40^{\circ }$, respectively, and the flapping frequency is 197 Hz for the honeybee.

## 3. Results

In order to illustrate the roles of resilin joints and stripes comparatively, four models were built, as follows: model 1 without resilin, model 2 with resilin joints, model 3 with resilin stripes, and model 4 with both of resilin stripes and resilin joints. The mechanical behaviors of the four models are studied and compared, using both static and dynamic analyses, in the following section.

### 3.1. Static Analysis of Flexibility

**Figure 3:**
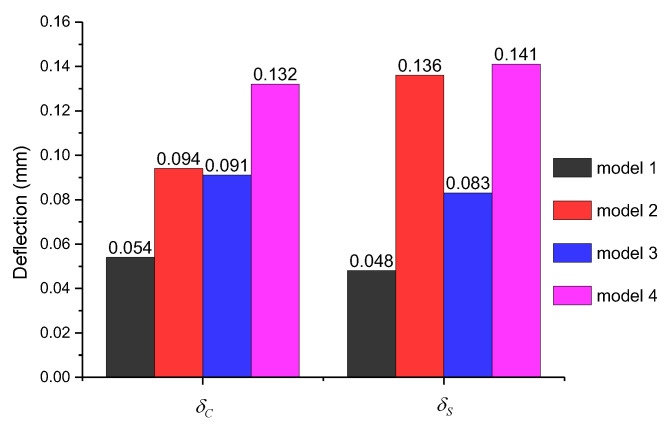
Deflections in chordwise and spanwise directions of the four models.

An experimental method to measure the chordwise stiffness of bumblebee wing was proposed by Mountcastle and Combes [10,13]. By fixing the location in the leading edge, the force exerted on the trailing edge is measured with a given deflection at 85% of chord length from the leading edge. Then, the chordwise stiffness can be calculated by the measured force and deflection. A similar approach is applied in our simulation. The leading-edge vein is fixed at the position of 50% of span length, and the deflection $\delta_{C}$ of a trailing-edge node is obtained when a concentrated force of 10^−5^ N is applied at about 85% of chord length in the trailing edge. $\delta_{C}$ was ensured to be less than 5% of the chord length in the simulation. In addition, the spanwise deflection $\delta_{S}$ is calculated in the same way, and the results of the four models are shown in Figure 3.

Both resilin joints and stripes have significant effects on wing flexibility from the comparisons of $\delta_{C}$ and $\delta_{S}$ through the four models. In the chordwise direction, $\delta_{C}$ is increased almost equally (by 70%) in model 2, with resilin joints, and in model 3, with resilin stripes. The effects double when both of them are used in model 4. The most striking increase in $\delta_{S}$ is in model 2, where it is 183% higher than that of model 1. Resilin joints have much more significant effects on spanwise flexibility, while resilin stripes have quite equal effects on them. Combining the two forms of resilin in model 4, the deflection of the wing is increased by about 1.4 times in the chordwise direction and 1.9 times in the spanwise direction.

**Figure 4:**
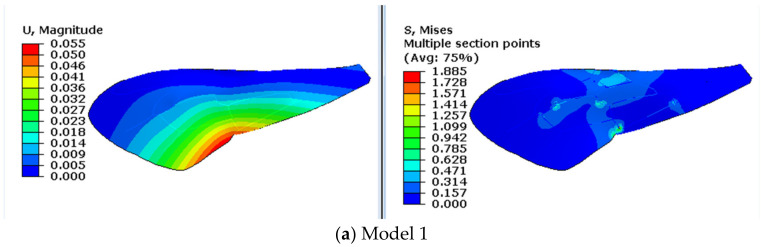

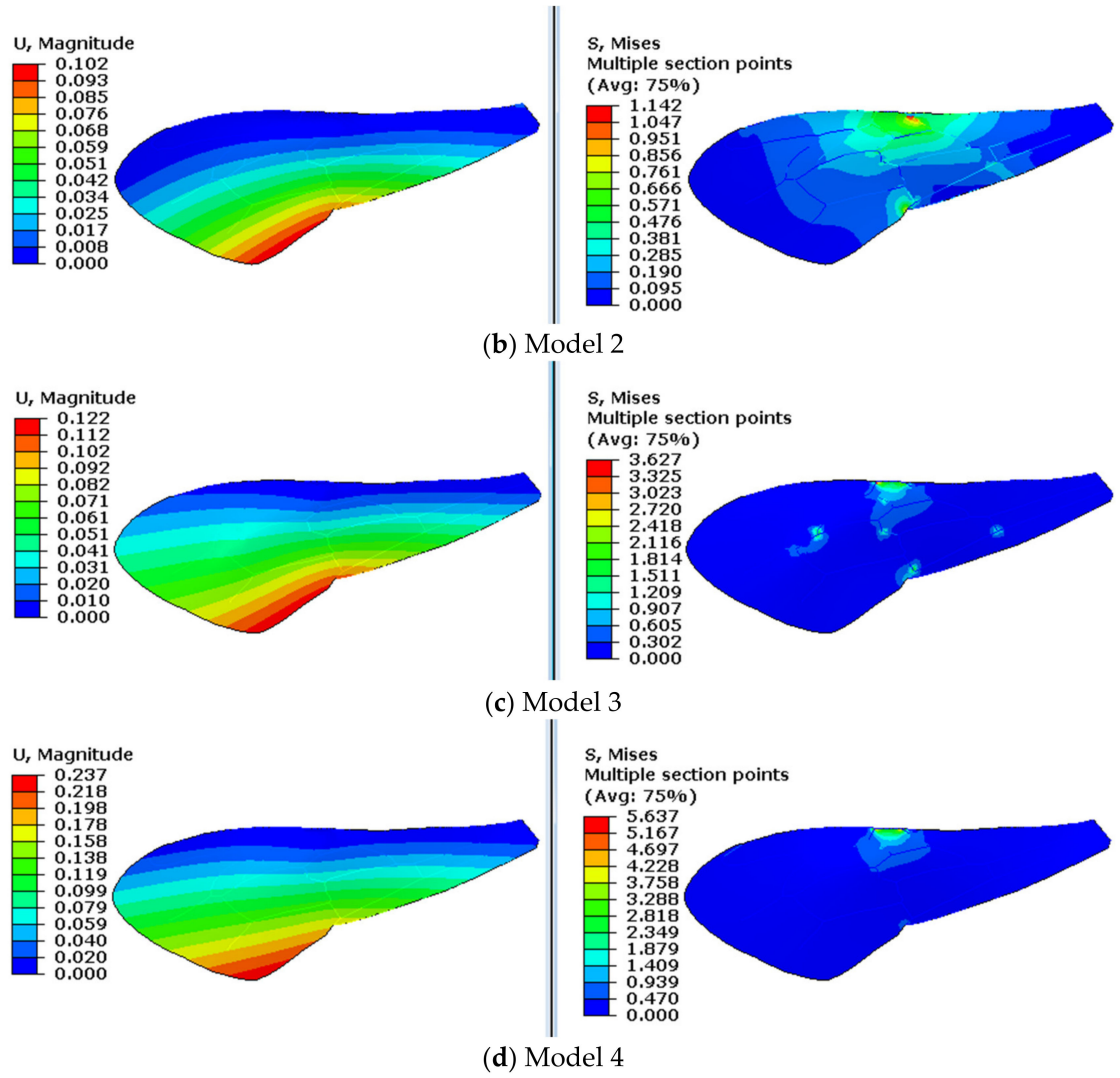
Chordwise deformation of the four models.

**Figure 5:**
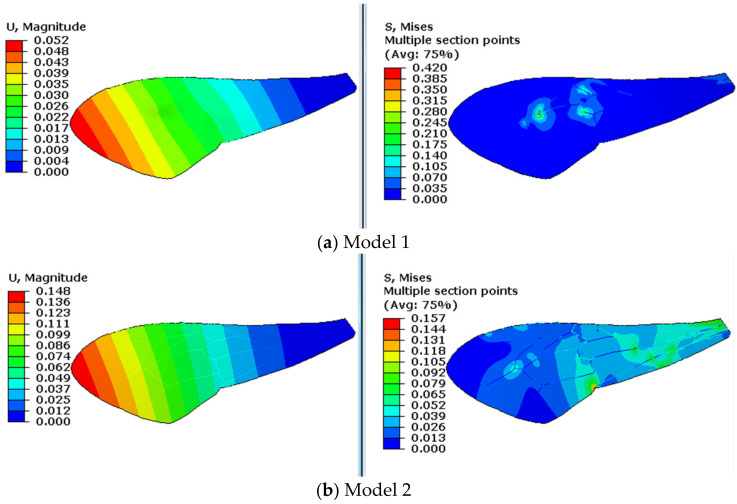

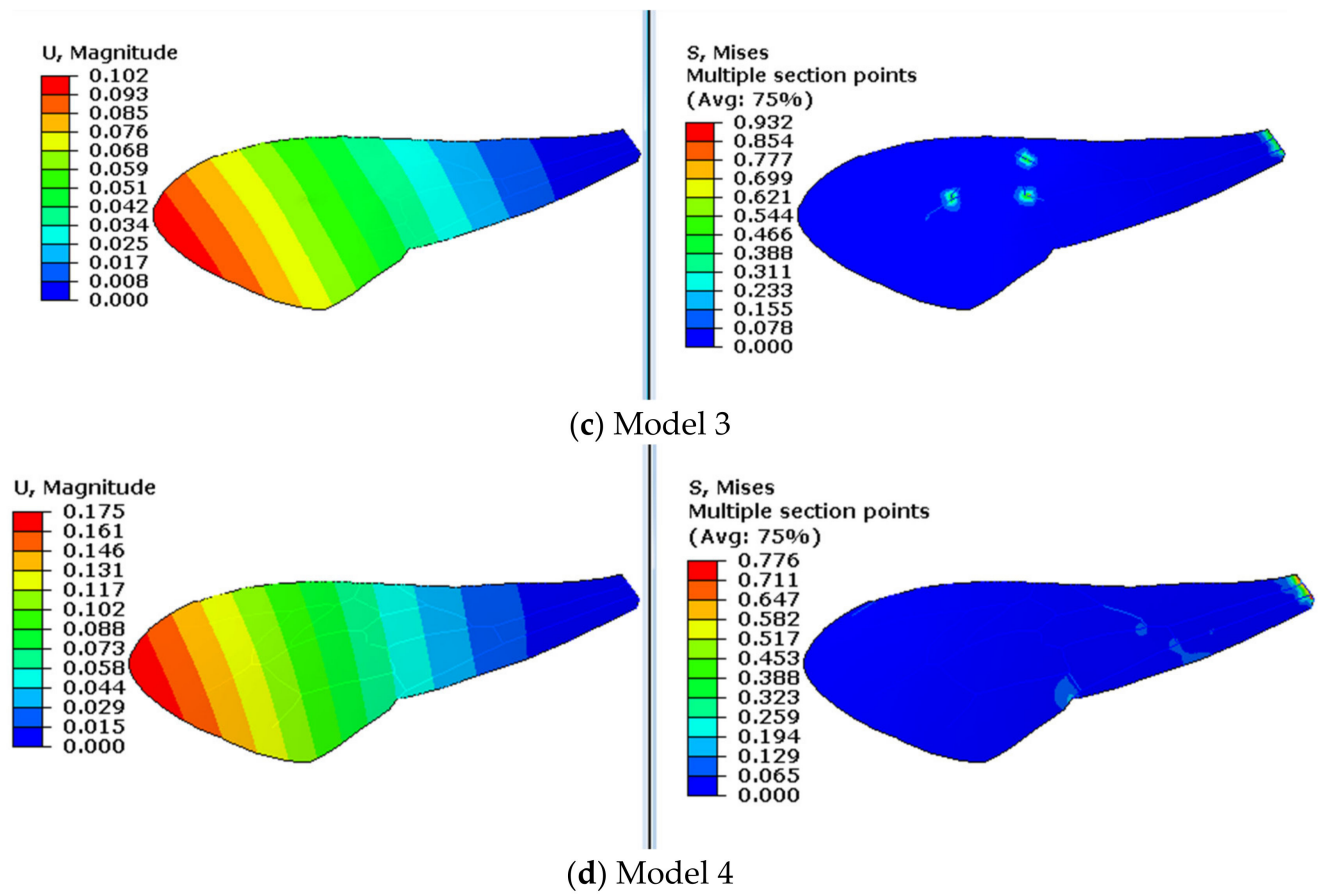
Spanwise deformation of the four models.

The contours of displacement and stress of the models are shown in Figure 4 and Figure 5. In chordwise deformation, displacement changes more gradually in the chord, and the stiffness along the chord decreases obviously when the resilin stripe is used in model 3 and model 4. As for the stress contours, stress concentrates along the vein stripes in model 2 and concentrates at the vein joints in model 3. When both resilin stripes and resilin joints are used, in model 4, these local stress concentrations disappear. The same situation was also observed in the stress contours in spanwise deformation, as shown in Figure 5. This illustrates that the coexistence of resilin joint and stripe can reduce local stress concentration in the middle areas of the wing in deformation. Considering the periodic deformation in flapping, this will benefit the fatigue resistance of the resilin-bearing areas.

Furthermore, this shows that the stress also concentrates near the end of anal vein, where the streamlined edge is curved. In fact, the forewing and hindwing are linked here by coiled membrane and a hook structure, achieving synchronous movement for honeybees. This special structure works with resilin to eliminate stress concentration, avoiding structural failure in flapping cycles [27,28].

### 3.2. Modal Analysis

The natural frequency is associated with the mass and stiffness of the wing. To understand the global stiffness of the models, modal analysis was carried out on the section. The total mass of the wing model was calculated as 130 mg, close to the experimental measurement of 102 mg [29]. With the base fixed, the first three modes and natural frequencies of the wing are extracted, as shown in Table 2.

The vibration modes of model 1 and model 3 are exactly the same, namely, the first-order bending mode, second-order bending mode, and first-order torsional mode. They differ only in that the frequencies of model 3 were lowered by 20–30% as resilin stripes were introduced. The first three vibration modes of model 2 and model 4 are first-order bending mode, second-order bending mode, and first-order torsional mode, which are different from that of model 1 and model 3. The most obvious difference is the frequency of the torsional mode, which was reduced from 500 Hz in model 1 to 239 Hz in model 2 and to 217 Hz in model 4. The results indicate that resilin joints have the most significant influences on rotational stiffness of a wing with a fixed base. The first bending frequency was decreased by about 20% in model 3, and by 40% in model 2 and model 4. As for the second-order bending mode, its frequency was least changed among the four models.

### 3.3. Dynamic Analysis of Flapping Flight

**Figure 6:**
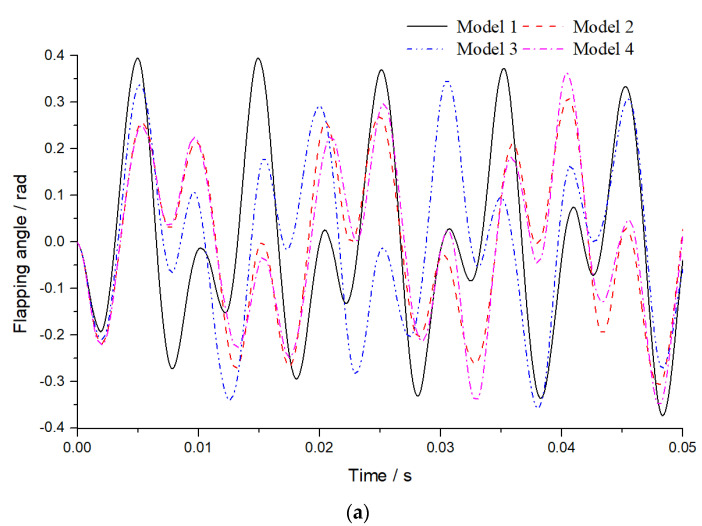

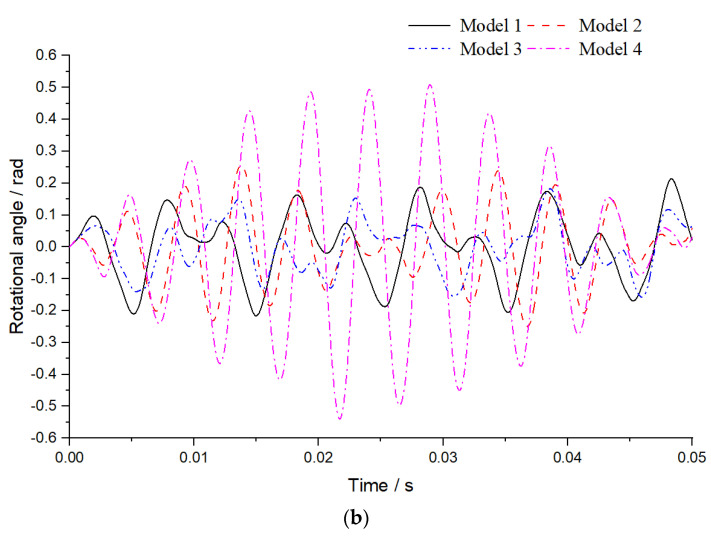
Dynamic responses of flapping angle (**a**) and rotational angle (**b**).

As flapping movement is driven by the basal muscles, flexibility and deformation will always influence the dynamic responses under the given basal motion. Hence, dynamic responses are simulated in this section to further study the roles of resilin. The flapping and rotational angles in Equation (1) are applied at the wing base, and the initial amplitudes are $\varphi_{\max}/10$ and $\alpha_{\max}/10$. The dynamic responses of flapping and rotational angles of a node in the trailing edge close to the wing tip are extracted, as shown in Figure 6.

The analysis time is 0.05 s, about 10 flapping periods. The frequency of the excitation is 197 Hz, falling in between the natural frequencies of the first-order bending mode and second-order bending mode for model 1 and model 3 and between the first-order bending mode and first-order torsional mode for model 2 and model 4. The base vibration mode is bending for all the four models. Therefore, the flapping angles shown in Figure 6a are considerable, especially for model 1 and model 3. The maximum flapping angle of model 1 is about $25^{\circ }$, while the amplitude applied on the base is only $6.5^{\circ }$. As the natural frequencies of the torsional mode are very close to the excitation frequency for model 4, the rotational angles in the dynamic responses are very remarkable, as shown in Figure 6b. The maximum rotational angle of model 4 is up to $32^{\circ }$, while the applied amplitude on the base is only $5^{\circ }$. Therefore, the amplitude amplification effect is significantly enhanced by resilin joints and stripes in torsion vibration.

## 4. Discussion

### 4.1. Comparison with Experiment

**Figure 7:**
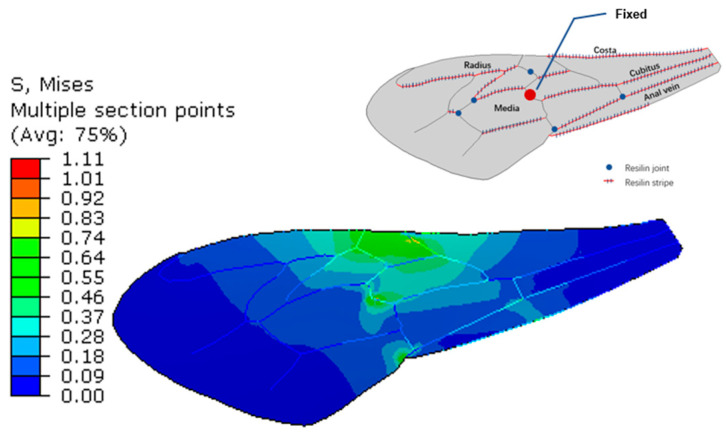
Stress contours of the wing with one resilin joint fixed.

The simulation shows that the six resilin joints increase the deflection in chordwise and spanwise directions by 74% and 183%, respectively. The chordwise stiffness of bumblebee forewing was found to be increased by more than 30% when one resilin-bearing joint located in the center was fixed artificially in the experiment [13]. To compare with these results, the resilin joint located in the same location was fixed in model 4, while the other five joints were set as hinged. The local stress concentration appeared around the fixed joint, as shown in Figure 7. The deflection of the trailing-edge node was obtained as 0.085 mm, while it was originally 0.132 mm, as shown in Figure 3. The value was decreased by 36% by the fixed vein joint. As the stiffness calculated in the experiment is linearly proportional to deflection when a central force is defined, the simulation is basically consistent with the experimental results. This verifies the accuracy of the finite element model in the analysis. Meanwhile, it also illustrates that the fixed joint in the center plays a fairly important role in the chordwise flexibility, as the deflection is just 0.091 mm when all the six joints are fixed as shown in Figure 3.

As vein corrugations have little effects on the chordwise stiffness, the simulation is basically consistent with experiment, though the small vein corrugations are not considered in the finite element models. The small differences between experiment and simulation occur because the diameter of the vein splint used in the experiment was 0.4 mm, which is much larger than the size of resilin joints in the finite element model. As for spanwise flexibility, the effects of resilin on deflection should be reconsidered when the pattern of vein corrugation which contributes to spanwise stiffness is introduced in the model. After all, the bending deformation of insect wings is restrained in flapping by the rigid and corrugated longitudinal veins. In aerodynamics, excessive spanwise bending can lower effective angles of attack and, consequently, lower aerodynamic force generation [30]. In addition, the efficiency of the flapping motion from the wing base to the tip plays a role in maintaining the spanwise stiffness.

### 4.2. Resilin Produces Camber Effects

**Figure 8:**
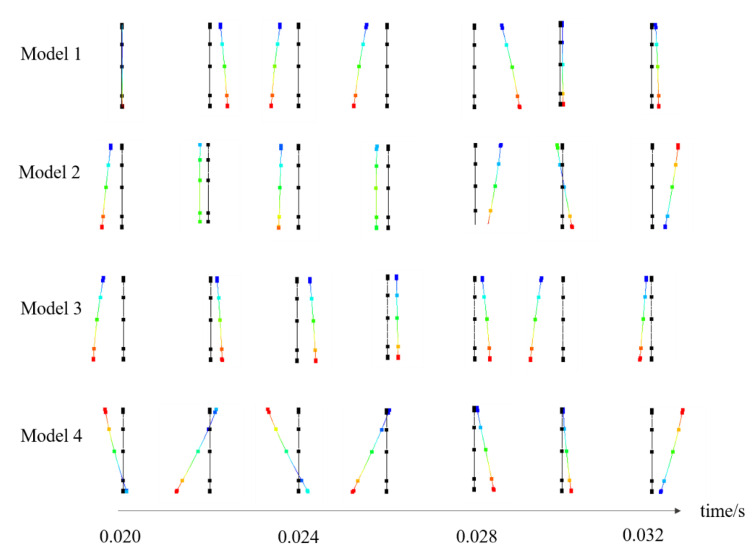
Cambered sections of honeybee forewing in dynamic flapping flight. The black straight line is the undeformed wing section and the colored is deformed.

In previous studies, it was found that the cambered sections of deformed wings of dragonflies and honeybees are related to resilin joints [19,31]. This umbrella effect on wing shape is considered as helping to improve the aerodynamics of upstrokes and downstrokes in flapping [5,32]. The wing cross-sections at half-chord length are extracted. Figure 8 shows how the shape of the section changes in dynamic flapping during the time of two periods. There are concave and convex sections in addition to the initial straight ones. All the sections of the four models are cambered to a certain extent, especially at the maximum flapping angle. For example, the wing flaps downward from 0.026 s to 0.028 s, during which the section changes from convex to concave. It changes in reverse during the upstroke of 0.028 s to 0.03 s. At the initial moments of upstrokes and downstrokes (supination and pronation), the cross-sections of the wing are always of an umbrella-like shape to meet the requirements of high lift [32]. The curved shape reverses in the following flap in order to produce drag reduction. The deformation is obtained without considering the aerodynamic load. Maximum rotation and camber could be observed in model 4 with both resilin joints and stripes under the driven motion on the wing base. This shows that the rotation and camber of the wing present in dynamic flapping are the combined effects of both resilin joints and stripes. Due to the material characteristics of resilin, there is no doubt that it will reduce wing stiffness when it appears. But the two different forms are believed to be an optimization of structure properties such as local deformation and stress distribution. In aerodynamic analysis, especially fluid-structure coupling analysis, the flexibility effect of resilin should be considered comprehensively.

### 4.3. Resilin Enhances the Rotation Efficiency

Driven by the basal muscle, insect wings are flapped at frequencies of tens to hundreds of times per second during flight. The input excitation from muscles is transmitted through the wing structure to produce the flapping motions. To satisfy the demands of aerodynamics, the flapping motion should always meet a certain amplitude and frequency to provide enough lift and thrust. It must be a formidable challenge for insects with only wing base connected with muscles if the wing is fully rigid. As the flexibility contributing factors, there is no doubt that the resilin joint and stripe will play roles in this dynamic process. Modal analysis shows that the natural frequency of the first torsional mode is dramatically reduced, by more than 50%, and more closely approaches the given frequency when resilin is introduced, especially resilin joints. This leads to a remarkable increase in rotational angle amplitude in dynamic responses.

To study the flexible roles of nodus in the flapping efficiency of dragonfly wings, the amplification ratio was defined as the ratio of displacements of the wing tip and wing base in a vibration test [33]. The same approach is used here to study the roles of resilin in the dynamic responses of rotational angle. With a given rotational amplitude of $5^{\circ }$ at the base, the rotational amplitude of model 4 in response is $32^{\circ }$. Therefore, the amplification ratio is 6.4, while that of model 1 is calculated as 1.5 without considering any resilin. When both resilin joints and stripes are used in the model, a great increase occurs. A certain rotational angle is necessary to improve the distribution of the angle of attack in the wingspan in aerodynamics. From the energy perspective, larger rotational amplitude means more energy consumption, as the rotation of the insect wing is considered to be applied to the wing base actively, aided by the inertial response [34]. The magnified effect of resilin on rotational angle amplitudes could help save flight energy consumption to some degree. In addition, the stress during the dynamics is always below 7 MPa, which is comparable with the static values of magnitude (see Figure 4 and Figure 5). Consequently, the active excitation of the muscles combined with the structural characteristics of both resilin joints and stripes can produce sufficient rotational amplitude. This could help enhance the efficiency of active rotation applied at the wing base.

## 5. Conclusions

The insect wing plays a very important part in transmitting motion and force during flapping and deformation. In this research, resilin joints and stripes, which have received much attention in recent years, are considered in the honeybee forewing by the finite element method. Their roles in determining overall wing stiffness and shaping wing deformations during flight are studied through both static and dynamic analysis. In static analysis, the six resilin joints have similar effects on chordwise flexibility to that of resilin stripes. Spanwise flexibility, by contrast, is influenced much more greatly by the six joints. Meanwhile, it was found that resilin stripes produce exactly the same effects on the two stiffnesses. In modal and dynamic analysis, resilin stripes result in a reduction of about 20–30% in each natural frequency, while resilin joints make a significant contribution, of up to 50%, to the reduction of torsional frequency. In dynamic analysis, together, they lead to the amplification of the amplitude of the dynamic rotational angle under base velocity by six times. Meanwhile, the wing sections are well cambered to benefit aerodynamics. This means that certain torsional angles and section shapes could be induced passively with resilin, due its effect on wing flexibility, to meet aerodynamic demands. In addition, it was found that the stress concentrating at the junction areas is improved when both forms of resilin are used in the models.

The results in the paper are based on the analysis of the finite element model of the honeybee forewing, with some simplifications. Although the actual irregular patterns of resilin on the wing are ignored, it is the first time that a study comprehensively considers the effects of both resilin joints and resilin stripes, and some meaningful conclusions are obtained. To obtain more accurate dynamic response behaviors of insect wings, the following improvements should be considered in future work. Firstly, the full coupling relationship of wing structure elasticity, inertia, and aerodynamics should be considered comprehensively. Secondly, the finite element model should be more accurate to be closer to the real wing. For example, the asymmetry of resilin in dorsal and ventral sides is well worth considering.

## Figures and Tables

**Table 1:** Materials and geometry parameters of the model. Vein ① is the costa, Vein ② are cubius and anal, Vein ③ and Vein ④ are radius and media in medium and trailing regions, respectively.

	Vein ①	Vein ②	Vein ③	Vein ④	Membrane
Outer diameter (μm)	40	30	20	10	
Thickness (μm)	10	7.5	5	2.5	6
Young’s modulus (GPa)	4.8				2.8
Poisson’s ratio	0.45				
Density (Kg/m^3^)	1200				

**Table 2:** Natural frequencies (Hz) and modes of the four models.

	The First	The Second		The Third	
	First-order bending 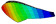	Torsion 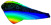	Second-order bending 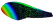	Second-order bending 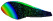	Torsion 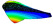
Model 1	100		339		500
Model 2	63	239		333	
Model 3	79		322		361
Model 4	62	217		322	

## Data Availability

Not applicable.
